# Trigeminal Trophic Syndrome Leading to Orbital Cellulitis

**DOI:** 10.5811/cpcem.2018.1.36622

**Published:** 2018-03-14

**Authors:** Linda B. Thompson, Stephen L. Powell

**Affiliations:** University of Alabama School of Medicine, Department of Emergency Medicine, Birmingham, Alabama

## Abstract

Trigeminal trophic syndrome is a rare condition that develops from trigeminal nerve damage causing dysesthesias that result in self-mutilation. Facial and nasal destruction develops from self-destructive behavior (repetitive picking or scratching) secondary to the altered skin sensation created by the damaged trigeminal nerve. Early recognition of this condition is crucial to the prevention of the detrimental complications of facial ulceration and nasal tissue necrosis that can lead to corneal ulcerations, full-thickness eyelid defect, and canthal lesions. This case demonstrates a previously unreported complication: orbital cellulitis.

## INTRODUCTION

Trigeminal trophic syndrome (TTS) is a rare condition that develops from trigeminal nerve damage causing dysethesias that result in self-mutilation.^1^,^2^,^3^ The nerve insult is frequently a result of surgery with subsequent anesthesia along the maxillary nerve branch of the trigeminal nerve (V_2_), but has also been reported in cases of alcohol injection of the trigeminal nerve used to treat trigeminal neuralgia, cerebral meningioma resection, cerebrovascular accidents, and herpes zoster ophthalmicus.^1^,^2^,^3^,^4^ This trigeminal neuropathy causes dysaesthetic skin and subsequently leads to compulsive pruritic sensations, self-mutilation, and eventual tissue necrosis.^1^,^2^,^3^,^4^ Case reports cite a characteristic “crescent”-shaped area of the nose lacking cartilage,^1^,^2^,^3^,^5^ or “persistent facial ulceration…with alar nasi involvement being a key feature.”^4^ Patients with TTS often have psychiatric comorbidities such as anxiety, obsessive-compulsive disorder (OCD), or mood dysfunction.^1^ Treatment is centered on preventing self-destruction with patient education and counseling.^2^,^3^,^4^ Reconstruction is typically unsuccessful due to further compulsive irritation.^4^,^6^,^7^ Though not previously described, our case demonstrates that orbital cellulitis can complicate TTS.

## CASE REPORT

A 75-year-old female with history of Type II diabetes presented to the emergency department (ED) with “double vision and right eye redness” for several weeks with isolated right eyelid swelling for one week. She had recently seen her ophthalmologist who noted the swelling and ordered magnetic resonance imaging (MRI), which showed inflammation extending from the right paranasal sinuses into the orbit. She was sent to the ED with concern for an infectious process such as mucormycosis. She had never had nasal, septal, or sinus surgery; however, she did have a remote history of trigeminal neuralgia treated with neurectomy 30 years prior. In the five years prior to this presentation, she had experienced facial paresthesias and pruritus managed by compulsive manipulation of her face, nose and sinuses with cotton tip applicators and fingers.

**Image 1:**
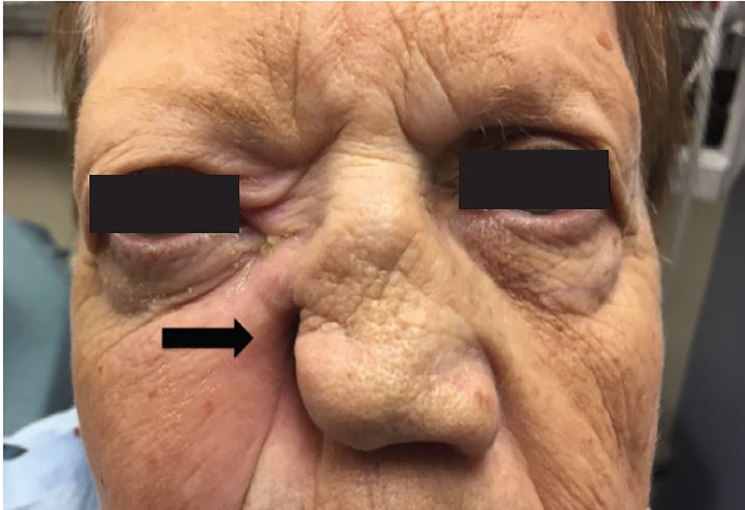
Right ala nasi erosive necrosis (arrow) with right orbital cellulitis and proptosis.

On physical exam, the patient was nontoxic with normal vital signs: temperature 98.1° F, heart rate 94 beats per minute, respiratory rate 18 breaths/min, blood pressure 121/72 mm/Hg. Visual acuity in each eye was 20/20 without visual aids. She was noted to have right eye proptosis, periorbital edema and erythema, in addition to right medial gaze palsy. She had erosive necrosis with complete destruction of the right ala nasi and grossly patent right nasal aperture. While the right inferior turbinate appeared normal, the right middle turbinate was scarred with extensive right septal scarring and scabbing (Image 1).

**Image 2:**
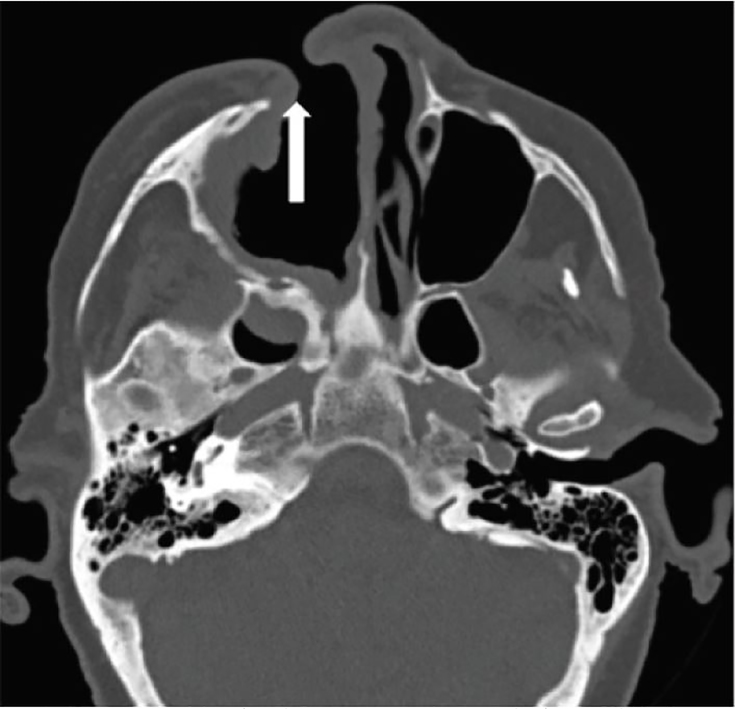
Axial computed tomography demonstrating anterior maxillary wall sinus erosion (arrow).

The patient’s laboratory testing revealed a white blood cell count 8.7 10^3^/cmm and serum glucose 216 mg/dl. Axial computed tomography (CT) (Image 2) showed dehiscence of the inferomedial right orbital wall with soft tissue density and stranding extending into the medial retro-orbital fat posterior to the orbital apex resulting in proptosis of the right eye. The right optic nerve sheath appeared thickened. The right nare was partially absent giving the appearance of a chronic wound or previous sinus surgery. Underlying encephalomalacia of the right temporal lobe from previous right neurectomy was also noted.

The patient was admitted with the diagnosis of orbital cellulitis and treated with parenteral vancomycin and piperacillin-tazobactam. Otolaryngology, ophthalmology, and psychiatry were consulted. Fiber optic endoscopy demonstrated extensive paranasal sinus destruction, with friable mucosa. The tissue destruction seen on CT and subsequent MRI was initially concerning for other serious infections, such as mucormycosis or malignancy; however, frozen pathology showed no evidence of an invasive fungal infection or malignant transformation. Other possible etiologies such as the autoimmune disorders Wegener’s granulomatosis or granulomatous polyangitis were considered.

**CPC-EM Capsule**

What do we already know about this clinical entity?

Trigeminal trophic syndrome (TTS) is a condition that follows trigeminal nerve damage resulting in compulsive pruritic sensations, self-mutilation, and eventual tissue necrosis.

What makes this presentation of disease reportable?

Orbital cellulitis resulting from TTS has not been reported in the literature; clinicians should be made aware of this potential serious complication.

What is the major learning point?

Early detection of TTS and recognition that treatment involves medical and psychiatric therapy is necessary to prevent devastating and disfiguring complications.

How might this improve emergency medicine practice?

Recognition of this disease and the potential serious complications will lead to the early, multidisciplinary management approach necessary for successful treatment, thus improving outcomes.

The required rheumatologic work up to evaluate for these potential causes would include the following: antinuclear antibody; C-reactive protein; erythrocyte sedimentation rate; and anti-neutrophil cytoplasmic antibodies. These lab tests were abandoned when the sinus culture was positive for *Staphylococcus aureus*, and the patient rapidly improved with the appropriate intravenous antibiotics. Psychiatric consultation determined that the patient had a component of an unrecognized OCD. The combination of ongoing facial paresthesia in the V_2_ division of the trigeminal nerve resulting from the previous trigeminal neuralgia surgery and the newly diagnosed OCD provided the diagnosis of TTS leading to the orbital cellulitis.

The patient was ultimately discharged on a course of oral amoxicillin-clavulanic acid and daily fluoxetine to address her previously undiagnosed obsessive-compulsive behavior. In a four-month clinic follow-up, the right ala nasi still appeared necrotic with the right nasal aperture still being widely patent. There was no overt evidence of active infection. The patient did not complain of any visual compromise and no visual acuity was obtained. The patient reported medication non-compliance and continued repetitive manual manipulation of her nose. She refused any further treatment.

## DISCUSSION

TTS is a rare condition that develops due to self-destructive behavior coupled with a damaged trigeminal nerve. The damaged trigeminal nerve most commonly is the result of trigeminal surgical ablation or alcohol injection for treatment of trigeminal neuralgia; however, it has been reported with cerebral meningioma resection, cerebrovascular accidents, herpes zoster ophthalmicus, vertebrobasilar insufficiency, acoustic neuroma, post-encephalitic parkinsonism, syringobulbia, meningioma, astrocytoma, Hansen’s disease, and trauma.^1^,^2^,^3^,^4^ The insulted trigeminal nerve leads to altered skin sensation such as numbness, tingling, and a pricking sensation in its dermatome.^1^,^2^,^3^,^4^ This altered skin sensation leads to self-induced trauma resulting in tissue destruction manifest as facial ulcerations and/or nasal cartilage destruction. The ala of the nose is a common location for tissue destruction given this area is a “subjectively intense focus of dysaesthesia due to the disparity in innervation at the junction of the trigeminal nerve braches: the ophthalmic nerve branch (V_1_) and the maxillary nerve brach (V_2_).”^4^

The differential diagnosis of patients with facial or nasal ulcerations can be extensive. Herpetic ulceration and other infectious diseases such as syphilis, yaws, leprous trigeminal neuritis, deep fungi, mycobacteria, rhinoscleroma, and leishmaniosis are among the possible infectious etiologies.^2^ Carcinoma, pyoderma gangrenosum and Wegener’s granulomatosis are also considerations.^2^ Though rare, TTS should also be included as a possible diagnosis.

The time period from trigeminal nerve injury to onset of the ulcer can be from weeks to several years, with a mean of 1–2 years.^2^ One review article indicates, “the neurotropic ulcerations may manifest weeks to years after the nerve damage, with up to a 20-year latency” with concerning complications including corneal ulcerations, canthal lesions and full thickness, upper eyelid defect.^4^ The management of TTS can be challenging. Patient education and counseling are a cornerstone of treatment.^4^ Occlusive dressings, topical lubricants, antibiotics, and surgical interventions may be considered; however, surgical interventions frequently fail if the physical manipulation of the skin is not controlled.^4^

Though not previously described, our case demonstrates that orbital cellulitis can also complicate TTS. In this case, the patient admitted to routine manual nose picking and repeatedly using a cotton swab to manipulate her nose for 4–5 years before presentation. This obsessive behavior led to the right alar necrosis and sinus erosion, an unlikely but direct etiology of the orbital cellulitis. Her history of diabetes most likely contributed to the severity of her infection. The management of this patient involved treating the orbital cellulitis with intravenous vancomycin and piperacillin-tazobactam. In an attempt to control her obsessive skin manipulation, she was started on fluoxetine. Sadly, the patient chose to discontinue her fluoxetine and return to self-mutilation, refusing any further attempts to improve her condition

## CONCLUSION

This case demonstrates a previously undescribed complication of trigeminal nerve injury in a patient with OCD. Trigeminal trophic syndrome is an important consideration when caring for patients with facial ulcerations and/or nasal destructive lesions who have a history of trigeminal nerve injury, even with a 30-year latency. Patients with psychiatric disorders and trigeminal neuralgia requiring surgical intervention, require pre-surgical counseling, extra vigilance in post-surgical follow-up, and consideration of appropriate psychiatric medications to prevent the complications of this syndrome.

Documented patient informed consent and/or Institutional Review Board approval has been obtained and filed for publication of this case report.
